# Why Narcissism Reduces Distress: The Consequences of Narcissistic Intellectual Self-Confidence

**DOI:** 10.3389/fpsyg.2021.668257

**Published:** 2022-02-03

**Authors:** Maria Leniarska, Marcin Zajenkowski

**Affiliations:** Faculty of Psychology, University of Warsaw, Warsaw, Poland

**Keywords:** admiration, distress, narcissism, rivalry, intelligence

## Abstract

The aim of the present study was to investigate the association between grandiose narcissism and the feeling of distress. We referred to the narcissistic admiration and rivalry model. We hypothesized that people with high narcissistic admiration would experience less distress and fear and that intellectual self-confidence would account for this relationship. We examined two dimensions of grandiose narcissism using Narcissistic Admiration and Rivalry Questionnaire, self-assessed intelligence, and various aspects of distress in two studies. In Study 1 (*N* = 170), we assessed distress (with the Dundee Stress State Questionnaire), related to performance in an intelligence test (Raven’s Advanced Progressive Matrices), and in Study 2 (*N* = 258) we measured fear related to the COVID-19 pandemic. In both studies, narcissistic admiration was inversely related to distress/fear, and this relationship was fully mediated by self-assessed intelligence. Narcissistic rivalry was unrelated to both distress and self-assessed intelligence. These findings emphasize the importance of self-views related to intelligence for those with high narcissistic admiration. In particular, intellectual self- confidence plays an important role in reducing distress among narcissists.

## Introduction

Narcissism has usually been studied in a negative context, such as psychopathology (e.g., Freud, 1914; Kohut, 1966) or in terms of antisocial personality traits (e.g., Paulhus and Williams, 2002). More recent findings have shown that narcissism may also be associated with positive outcomes, such as intrapersonal adjustment (e.g., Dufner et al., 2019). Narcissists’ positive or negative characteristics might, however, depend on the specific type. According to a widely accepted distinction, narcissism has two major forms: grandiose and vulnerable (Wink, 1991; Miller et al., 2011; Krizan and Herlache, 2018). Their common core is self-centeredness and a sense of entitlement, but they differ in many respects (Krizan and Herlache, 2018). Vulnerable narcissism is related to withdrawal, low self-esteem, negative affect, sensitivity to negative feedback, and defensiveness (Wink, 1991). Grandiose narcissism reflects positive self-esteem, inflated self-views, high approach motivation, social confidence, and the need for admiration (Campbell and Miller, 2011). Another conceptualization distinguishes two dimensions within grandiose narcissism: narcissistic narcissistic admiration and narcissistic rivalry. This is the narcissistic admiration and rivalry concept (NARC; Back et al., 2013). Both narcissistic admiration and narcissistic rivalry reflect the narcissistic need to sustain grandiose self-views, but they achieve this through different strategies (Back et al., 2013). Narcissistic admiration is characterized by a seeking for achievement, social dominance, a desire for self-uniqueness, high self-esteem, agentic behavior, and grandiose fantasies (Back et al., 2013; Rogoza et al., 2016a,b, 2019). By contrast, narcissistic rivalry is related to lower self-esteem, disagreeableness, anger, and impulsivity (Back et al., 2013; Rogoza et al., 2016b,Rogoza et al., 2019), and is characterized by antagonism and the devaluing of others’ behavior (Back et al., 2013). Grandiose narcissism has been linked to positive outcomes, mainly in the area of intrapersonal processes (Czarna et al., 2018). It was found to correlate with a wide range of indicators of psychological adjustment, such as high levels of life satisfaction, positive affect, relationship satisfaction, and low levels of anxiety, sadness, depression, and loneliness (Sedikides et al., 2004). Further analysis has revealed a crucial role of high self-esteem, which mediated the relationship between grandiose narcissism and all aspects of psychological adjustment (Sedikides et al., 2004). Sedikides et al. (2004) suggested that this narcissistic self-esteem may derive from the perceived competence and feelings of agency observed among grandiose narcissists. In line with this view, it was found that grandiose narcissism was positively related to mental toughness (Sabouri et al., 2016), which is closely associated with self-confidence and a sense of control over one’s life (Clough et al., 2002), and that mental toughness is responsible for reduced depression and stress among grandiose narcissists (Papageorgiou et al., 2019). However, narcissistic self-confidence might stem from overly optimistic self- views (Zajenkowski et al., 2020a) and a biased perception of feedback, that is, the tendency to interpret neutral information as rather positive and to ignore critical feedback (Meisel et al., 2016; Howes et al., 2020).

Empirical findings and well-known models of narcissism suggest that agency is indeed an important source of high self-esteem in grandiose narcissism (Campbell and Foster, 2007; Morf and Rhodewalt, 2001). Among various agentic attributes, intelligence is regarded as one of the most prototypical (Abele and Wojciszke, 2014). Self-perception of intelligence is regarded as a specific form of Bandura (2001) self-efficacy (Howard and Cogswell, 2018). Self-assessed intelligence is an indicator of one’s cognitive abilities, which became essential to modern society. Thus, it has been suggested that, nowadays, SAI determines people’s self- worth (Howard and Cogswell, 2018). Considering the above, intelligence should be of high value for grandiose narcissists. Research findings support the view that intelligence plays a central role in grandiose narcissists’ lives (Zajenkowski and Dufner, 2020). Grandiose narcissism is one of the strongest predictors of self-assessed intelligence (Howard and Cogswell, 2018) and of overestimation of one’s cognitive ability (Zajenkowski et al., 2020a). Intellectual self-confidence also lowered the level of distress experienced when solving an IQ test (Zajenkowski et al., 2020a). A more fine-grained study revealed that one dimension of grandiose narcissism is associated especially with self-assessed intelligence: narcissistic admiration (Zajenkowski et al., 2020c). Furthermore, people scoring high on narcissistic admiration believed that intelligence was an important factor in determining one’s popularity, and their inflated self-views on intelligence partially explained their increased life satisfaction (Zajenkowski et al., 2020c).

As mentioned above, grandiose narcissism is associated with higher well-being (Sedikides et al., 2004; Dufner et al., 2019) and lower perceived stress (Ng et al., 2014). Moreover, there is evidence that this intrapersonal adjustment might stem from better coping ability (i.e., stopping ineffective coping strategies and adopting adaptive ones; Ng et al., 2014). In line with this finding is the research showing that grandiose narcissists display higher resilience (Sękowski et al., 2021). Specifically, grandiose narcissism is relatively highly associated with ecological resilience and adaptive capacity (Sękowski et al., 2021). Ecological resilience is the ability to resist disturbances typically accompanied by self- confidence in one’s strengths, while adaptive capacity reflects the ability to adapt to stressful situations, unstable conditions, or accommodate to the change (Maltby et al., 2015). Thus, grandiose narcissists show higher levels of agentic aspects of resilience which allow them to manage stress and recover after experiencing stressful events (Sękowski et al., 2021).

### Current Research

Grandiose narcissists display a high need for agency (Campbell and Foster, 2007). A feeling of control, confidence, and agency is the basis of their self-esteem, which in turn leads to increased well-being and psychological adjustment (Sedikides et al., 2004). Intelligence, a highly agentic characteristic, is thus crucial for grandiose narcissists’ self-concept (Zajenkowski and Dufner, 2020). The present study examines the consequences intellectual self-confidence has in the regulation and reduction of psychological distress among grandiose narcissists.

In the two studies presented herein we referred to the narcissistic admiration and rivalry concept, expecting that self-assessed intelligence would be correlated (positively) with narcissistic admiration only (H1). We also examined the potential consequences of self-views of intelligence. In Study 1, we focused on a domain corresponding to the content of these beliefs by investigating how narcissists would feel when trying to solve an IQ test. We expected that high levels of narcissistic admiration would be associated with lower distress (H2a) and that self-assessed intelligence would mediate this relationship (H2b).

In Study 2, we were interested to discover whether intellectual self-confidence among those with high narcissistic admiration generalizes to other (than intelligence testing) areas. In particular, we tested whether beliefs about intelligence would help narcissists to cope with the difficult situation of the COVID-19 pandemic. Prior findings indicate that grandiose narcissists display high levels of coping flexibility (Ng et al., 2014) and resilience (Sękowski et al., 2021). It has been suggested that their ability to adapt to demanding, unstable conditions might be rooted in their self-confidence and sense of agency (Sękowski et al., 2021). Thus, grandiose narcissists should cope well with the unexpected and demanding situation of the COVID-19 pandemic. Because narcissistic admiration seems to be a more representative aspect of grandiosity and agency (Rogoza et al., 2019), we expected that narcissistic admiration would be associated with lower fear (H3a) and that self-assessed intelligence would mediate this relationship (H3b).

## Study 1

We examined the association between two aspects of grandiose narcissism (i.e., narcissistic admiration and rivalry) and intelligence. We assessed these objectively with an IQ test and subjectively with a self-report measure. In addition, we examined whether narcissism and intelligence were associated with stress states during the performance of the IQ test. We applied the concept of task-related stress (Matthews et al., 2002), which distinguishes between motivational, affective, and cognitive aspects of subjective stress experienced during cognitive performance. Matthews et al. (2002) described three broad factors of stress states: (a) task engagement, integrating task interest, energy, motivation, and concentration; (b) distress, reflecting negative affect, tension, and lack of confidence; and (c) worry, referring to cognitive processes such as task-irrelevant thoughts and self-focused attention.

### Method

#### Participants and Procedure

A total of 170 participants (67.1% women, 31.8% men, and 1.2% other) took part in an online study, which was distributed via social media (e.g., Facebook). The link to the study was posted on various Polish groups from social network websites. Their ages ranged from 19 to 56 (*M* = 24.75, *SD* = 6.78). All had Polish nationality; 33% had a university degree, 62% were university students, and 5% had a secondary education. They were provided with information about the aim of the research before completing a set of questionnaires and tasks. Each participant gave informed consent.

#### Measures

Narcissism was assessed using the Polish version (Rogoza et al., 2016a) of the Narcissistic Admiration and Rivalry Questionnaire (Back et al., 2013). The scale includes nine items for each of the two dimensions, which are admiration (e.g., “I show others how special I am”) and rivalry (e.g., “I enjoy it when other people are inferior to me”). Participants were asked how much they agreed with the statements on a six-point scale (1 = *not agree at all*; 6 = *agree completely*). Items for each scale were averaged to obtain the relevant indexes. The internal consistency of narcissism subscales was high (Admiration α = 0.85; Rivalry α = 0.75).

Self-assessed intelligence was estimated using Zajenkowski and Gignac (2018) methodology. Participants were asked to estimate their intelligence compared with other people, in a scale from 1 (*very low*) to 25 (*very high*). The scores were standardized and transformed to IQ scores (*M* = 100, *SD* = 15).

Objective intelligence was measured using the Advanced Progressive Raven Matrices (Raven et al., 1983). This is a widely used non-verbal measure that captures mainly the fluid aspect of intelligence. We used a short version consisting of 18 items.

Task-related stress states were assessed using a Polish version (Zajenkowski and Matthews, 2019) of the Dundee Stress State Questionnaire (DSSQ; Matthews et al., 2002). The DSSQ measures three dimensions of states experienced during task performance: task engagement (“I was focused on the task”), distress (“I think the task was too difficult for me”), and worry (“I was thinking about my problems and matters”). The scale contains 24 items, eight items for each subscale, with five-point response scales (0 = *definitely false*; 4 = *definitely true*). Participants were asked to complete the DSSQ immediately after performing Raven’s test. All subscales had acceptable internal consistency (Task engagement α = 0.79; Distress α = 0.60; Worry α = 0.67).

### Results

We present correlations and descriptive statistics in Table 1. Narcissistic admiration and narcissistic rivalry were positively correlated. Self-assessed intelligence was positively correlated with narcissistic admiration and negatively with narcissistic rivalry. Narcissistic admiration and self-assessed intelligence were negatively correlated with distress related to test performance. Objective intelligence was positively correlated with self-assessed intelligence and task engagement, and negatively with worry.

**TABLE 1:** Correlations between narcissism, intelligence, self-assessed intelligence, and descriptive statistics.

	*M*	*SD*	1	2	3	4	5	6
1. Narcissistic admiration	3.29	0.86						
2. Narcissistic rivalry	2.71	0.78	0.31**					
3. Objectively assessed intelligence	17.78	2.83	–0.08	–0.04				
4. Self-assessed intelligence	100.00	15.00	0.39**	0.07	0.1*			
5. Task engagement	2.47	0.70	–0.00	–0.04	0.36**	0.11		
6. Distress	2.07	0.55	−0.19*	–0.08	–0.07	−0.25**	–0.10	
7. Worry	1.58	0.70	0.10	0.05	–15*	–0.09	–0.05	0.39**

*p < 0.05, **p < 0.01.

**FIGURE 1:**
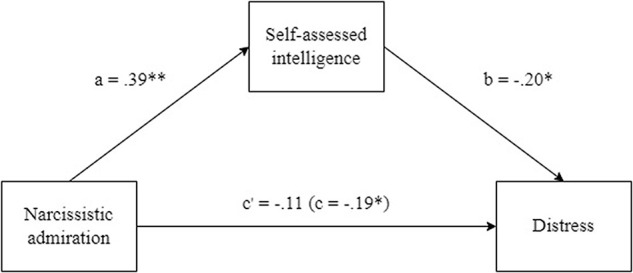
Relationship between narcissistic admiration, distress and self-assessed intelligence. The paths with a and b are direct, c is total effect from narcissistic admiration to distress and c′ is the direct effect, controlling self-assessed inteligence. ^∗^*p* < 0.05, ^∗∗^*p* < 0.01.

Subsequently, we tested whether the relationship between narcissistic admiration and distress was mediated by self-assessed intelligence (see Figure 1). The analysis was conducted using the PROCESS macro for SPSS (Hayes, 2015). The mediation was performed on standardized scores, using bootstrapping (5,000) and a 95% confidence interval (CI). We found that the indirect effect of self-assessed intelligence was significant (β = −0.08, *p* < 0.05, 95% CI [−0.15, −0.02]). The effect of narcissistic admiration on distress (β = −0.19, *p* < 0.05) decreased when the mediator self-assessed intelligence was included (direct effect: β = −0.11, *p* > 0.05). In conclusion, the relationship between narcissistic admiration and distress when performing the task was fully mediated by self-assessed intelligence.

### Discussion

The results of Study 1 showed a positive relationship between narcissistic admiration and self-assessed intelligence, therefore confirming H1 and replicating previous findings (Zajenkowski et al., 2020c). Narcissistic admiration was also inversely related to task performance distress, therefore confirming H2a and replicating previous findings (Sabouri et al., 2016). Further analysis revealed that the inverse relationship between narcissistic admiration and distress was mediated by self-assessed intelligence, so H2b was confirmed. The results indicated that narcissistic self-confidence related to intelligence played an important role in regulating negative affect in the context of performing a demanding task. However, Study 1 examined distress in response to an IQ test, a situation that was directly linked to self-assessed intelligence (i.e., a belief that one is intelligent might reduce distress in the situation that requires intelligence). A further question would be: To what extent do the effects of narcissistic intellectual self-confidence generalize to other life domains? We tested this in Study 2.

## Study 2

In Study 2, we examined associations between two aspects of grandiose narcissism (admiration and rivalry), self-assessed intelligence, and fear related to the COVID-19 pandemic. We included several questions relating to the perceived risk of infection, fear about one’s health, and fear about one’s future.

### Method

#### Participants and Procedure

A sample of 258 volunteers (28.3% male, 70.9% female, and 0.8% non-declared) of Polish nationality participated in an online study, which was distributed via social media (e.g., Facebook). The link to the study was posted on various Polish groups from social network websites. Their ages ranged from 18 to 80 (*M* = 28.96, *SD* = 10.69); 49% of them had a university degree. Before participating, they were given information about the general aim of the study, and were asked to sign the appropriate agreements. They then completed a series of questionnaires. The data were collected during the first wave of the COVID-19 pandemic and the lockdown in Poland (April 14–20, 2020). During this time, the government set down strict restrictions limiting social contact (e.g., isolation from people outside the household, restrictions on movement and travel, limitations in the number of customers allowed in shops, and closures of schools and restaurants) and encouraged people to work from home. Study 2 was a part of a larger project (see Zajenkowski et al., 2020b).

#### Measures

Narcissism and self-assessed intelligence were estimated using the same measures as Study 1. The internal consistencies of the narcissism subscales were high (Admiration α = 0.74; Rivalry α = 0.81).

Fear related to COVID-19 was estimated by asking participants about the risk of infection (“What is the risk that you will become infected?”); fear concerning health (“To what extent do you fear for your own health?”); and fear about the future (“To what extent do you fear what will happen next?”). Participants responded to each question on a scale from 1 (*not at all*) to 100 (*very much*).

### Results

In Table 2, we present correlations and descriptive statistics of all variables. Narcissistic admiration and rivalry were positively correlated. Narcissistic admiration was negatively associated with fear about one’s health and fear about the future, whereas narcissistic rivalry was not associated with any aspect of the COVID-19 situation. Self- assessed intelligence was positively associated with narcissistic admiration and negatively with fear about the future.

**TABLE 2:** Correlations between narcissism, self-assessed intelligence, perception of COVID-19 situation and descriptive statistics.

	*M*	*SD*	1	2	3	4	5
1. Narcissistic admiration	3.31	0.85					
2. Narcissistic rivalry	2.51	0.76	0.14*				
3. Self-assessed intelligence	100.00	15.00	0.31**	0.06			
4. Risk of infection	37.67	24.45	–0.03	0.00	0.00		
5. Fear about ones health	34.92	27.35	−0.13*	–0.06	–0.06	0.38**	
6. Fear about future	61.50	30.75	−0.13*	–0.02	−0.15*	0.13*	0.43**

*p < 0.05, **p < 0.01.

**FIGURE 2:**
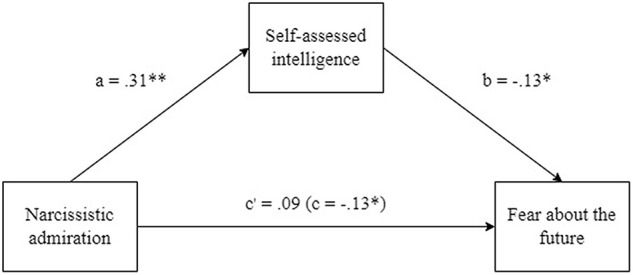
Relationship between narcissistic admiration, fear about the future and self-assessed intelligence. The paths with a and b are direct, c is total effect from narcissistic admiration to fear about the future and c′ is the direct effect, controlling self-assessed inteligence. ^∗^*p* < 0.05, ^∗∗^*p* < 0.01.

We then tested the mediating effect of self-assessed intelligence on the relationship between narcissistic admiration and fear about the future (see Figure 2). The mediation was performed using the PROCESS macro for SPSS (Hayes, 2015). The mediation was based on standardized scores, using bootstrapping (5,000) and a 95% confidence interval. The indirect effect of self-assessed intelligence was significant (β = −0.04, *p* < 0.05, 95% CI [−0.084, −0.002]. The effect of narcissistic admiration on fear about the future (β = −0.13, *p* < 0.05) was substantially reduced (direct effect: β = −0.09, *p* > 0.05) upon the inclusion of self- assessed intelligence. Thus, the relationship between narcissistic admiration and fear about the future was fully mediated by self-assessed intelligence.

### Discussion

In Study 2 we replicated the positive relationship between narcissistic admiration and self-assessed intelligence, thus confirming H1. Narcissistic admiration was negatively correlated with fear related to the COVID-19 pandemic (i.e., concerning health and the future), which confirmed H3a. However, self-assessed intelligence was negatively associated with fear about the future only, and mediated the relationship between admiration and the latter.

## General Discussion

We examined the association between two aspects of grandiose narcissism (admiration and rivalry), a feeling of distress, and self-assessed intelligence. We found across the two studies that narcissistic admiration was correlated with a low level distress and a high level of self-assessed intelligence. Additionally, intelligence self-views mediated the relationship between admiration and distress.

Our findings are in line with previous studies showing that grandiose narcissism correlates with a high level of well-being and a low level of negative emotionality (Sedikides et al., 2004). However, we expanded on those results by showing that narcissistic admiration reduced negative feelings of distress and fear, whereas narcissistic rivalry was unrelated to affective experiences. Although both narcissistic admiration and rivalry are considered subdimensions of grandiose narcissism (Back et al., 2013), there is evidence that they might be differently located in the narcissism spectrum that distinguishes grandiosity and vulnerability (Krizan and Herlache, 2018). Narcissistic grandiosity is typically linked to positive emotions (Sedikides et al., 2004), while vulnerability is associated with negative emotionality such as anxiety, depression, tension, and anger (Wink, 1991; Miller et al., 2011). Narcissistic rivalry shares certain characteristics with both narcissistic admiration and vulnerable narcissism, and is located between grandiosity and vulnerability (Rogoza et al., 2018). This duality might explain the lack of correlation with distress in the present study. We found that self-assessed intelligence mediated the relationship between admiration and distress. In Study 1, people with high narcissistic admiration estimated their intelligence to be high, which in turn led to lower level of distress in performing the IQ test. We found a similar effect in Study 2, though in this case we examined fear about the future in the context of the COVID-19 pandemic. Again, positive intelligence self-views resulted in less fear among those with high admiration. The results of our studies are consistent with findings pointing to the importance of agentic attributes—such as intelligence—in grandiose narcissists’ inner lives (Zajenkowski and Dufner, 2020). The belief that one is intelligent is not only a source of positive (or less negative) feelings in the domain directly associated with intelligence (i.e., IQ test performance); it also generalizes to other areas (e.g., fear about the future during a pandemic). For narcissists, however, intelligence may play a substantial role beyond these domains. Many theoretical models treat grandiose narcissism as a self-regulatory system consisting of various traits, abilities, emotions, beliefs, strategies, and behavior that interact with and mutually reinforce each other (e.g., Campbell and Foster, 2007; Morf and Rhodewalt, 2001). For instance, the extended agency model assumes that narcissists’ inflated views of their intelligence may reinforce beliefs about their superiority, which may consolidate social confidence and in turn reinforce their positive views of their intelligence (Campbell and Foster, 2007). Therefore, inflated self-views in an agentic domain such as one’s IQ have an impact on different areas, and serve a self-regulatory function in maintaining positive feelings and high self-esteem. Additionally, recent findings suggest that the positive consequences of narcissistic admiration might be even more pronounced among those who are actually high on objective intelligence. Specifically, Gignac and Zajenkowski (2021) have found that a high level of objectively measured intelligence reduced the chance of developing narcissistic rivalry. This would suggest that not only self-assessed but also objective IQ might be beneficial for those scoring high on narcissistic admiration.

An important question is whether emotions experienced by people with high narcissistic admiration might have positive behavioral consequences. As we have already mentioned, grandiose narcissists’ emotional experiences are rooted in the positive picture they have of themselves (Campbell and Foster, 2007). However, this self-image is often unrealistic and overly positive (Grijalva and Zhang, 2015). Their feelings, which are based on false perception of their abilities, may therefore have little impact in real life. For instance, the lower distress related to IQ test performance among people with high narcissistic admiration observed in Study 1 did not result in a better score. Likewise, it is possible that low fear about the future (Study 2) will not translate into actual behavior. This expectation is in line with findings showing that grandiose narcissism leads to intra-personal adjustment; in the context of inter-personal relations, it can create difficulties, especially in long term relationships (Czarna et al., 2016; Foster and Brunell, 2018).

It is worth noting that the overly positive self-views might also have negative consequences for narcissistic individuals. It has been found that narcissists’ self-concept is fragile and they show pronounced defensive reactions when their intelligence is threatened. For instance, their self-esteem decreases when they receive negative feedback on their IQ (Rhodewalt and Morf, 1998) and that they respond aggressively when their ability is called into question (Bushman and Baumeister, 1998).

## Limitations

The present study extended previous findings by demonstrating the existence of an inverse relationship between narcissistic admiration and distress, which was mediated by self-assessed intelligence. Nonetheless, our research is not free of some limitations. First, although we have shown that self-assessed intelligence reduces fear among narcissists in a domain other than IQ testing, more research is needed to establish whether intellectual self- confidence generalizes to other domains, for instance, school achievements or job performance. Second, while we had generally good internal consistencies for our scales (i.e., αs ≥ 0.70; Nunnally, 1978), some had only fair properties (i.e., αs ≥ 0.50; Schmitt, 1996). For instance, the distress subscale from the DSSQ had relatively low reliability (α = 0.60). Thus, future studies might consider using other scales to replicate our findings. Third, we relied on single item measures of fear related to various aspects of the COVID-19 pandemic which may reduce measurement quality. Nevertheless, we think that this specific type of fear was sufficiently salient to people that a multi-item scale was not needed. Moreover, our results are consistent with previous research, concerns over single-item assessments seem to be minimized here. Fourth, in both studies we used convenience sampling as we distributed the surveys via social media. Future studies might replicate our findings on more representative samples. Fifth, the mediating role of SAI in the relationship between admiration and distress was relatively small. Arguably, other factors might account for narcissists’ lower distress, such as self-confidence in other than intellectual domain (e.g., social), resilience or coping strategies (see Sękowski et al., 2021). Sixth, we found relatively low correlations between admiration and rivalry scales in comparison to the original study by Back et al. (2013). A recent study revealed that there might exist factors (e.g., the level of intelligence) that moderate the association between narcissistic admiration and rivalry (Gignac and Zajenkowski, 2021), which might explain the fluctuation of correlations across studies.

Finally, we took into consideration only self-perceptions of intelligence as an aspect of narcissistic self-confidence. However, self-confidence is a complex construct that is contingent on many other factors. Future studies could examine other than intelligence-related beliefs that help grandiose narcissists to reduce negative emotions. Moreover, because our research was correlational, we could only speculate about processes associated with emotion regulation in narcissism. Thus, experimental studies might shed more light on the role of self-confidence in the self-regulatory processes among grandiose narcissists.

## Data Availability Statement

The raw data supporting the conclusions of this article will be made available by the authors, without undue reservation.

## Ethics Statement

Ethical review and approval was not required for the study on human participants in accordance with the local legislation and institutional requirements. The patients/participants provided their written informed consent to participate in this study.

## Author Contributions

ML and MZ developed the idea of the manuscript. ML drafted the manuscript and conducted the analyses. MZ reviewed and edited the final version of the manuscript. Both authors contributed to the article and approved the submitted version.

## Conflict of Interest

The authors declare that the research was conducted in the absence of any commercial or financial relationships that could be construed as a potential conflict of interest.

## Publisher’s Note

All claims expressed in this article are solely those of the authors and do not necessarily represent those of their affiliated organizations, or those of the publisher, the editors and the reviewers. Any product that may be evaluated in this article, or claim that may be made by its manufacturer, is not guaranteed or endorsed by the publisher.

## References

[B1] Abele A. E. Wojciszke B. (2014). Communal and agentic content in social cognition: a dual perspective model. *Adv. Exp. Soc. Psychol.* 50 195–255.

[B2] Back M. D. Küfner A. C. P. Dufner M. Rauthmann J. F. (2013). Narcissistic admiration and rivalry: disentangling the bright and dark sides of narcissism. *J. Pers. Soc. Psychol.* 105 1013–1037. 10.1037/a0034431 24128186

[B3] Bandura A. (2001). Social cognitive theory: an agentic perspective. *Annu. Rev. Psychol.* 52 1–26. 10.1146/annurev.psych.52.1.1 11148297

[B4] Bushman B. J. Baumeister R. F. (1998). Threatened egotism, narcissism, self-esteem, and direct and displaced aggression: does self-love or self-hate lead to violence? *J. Pers. Soc. Psychol.* 75:219. 10.1037//0022-3514.75.1.219 9686460

[B5] Campbell W. K. Foster J. D. (2007). “The narcissistic self: background, an extended agency model, and ongoing controversies,” in *Frontiers of Social Psychology: The Self*, eds Sedikides C. Spencer S. J. (New York, NY: Psychology Press), 115–138.

[B6] Campbell W. K. Miller J. D. (2011). *Handbook of Narcissism and Narcissistic Personality Disorder: Theoretical Approaches, Empirical Findings, and Treatments.* Hoboken, NJ: Wiley.

[B7] Clough P. Earle K. Sewell D. (2002). “Mental toughness: the concept and its measurement,” in *Solutions in Sport Psychology*, ed. Cockerill I. M. (London: Cengage Learning), 32–43.

[B8] Czarna A. Z. Leifeld P. Śmieja M. Dufner M. Salovey P. (2016). Do narcissism and emotional intelligence win us friends? modeling dynamics of peer popularity using inferential network analysis. *Pers. Soc. Psychol. B* 42 1588–1599. 10.1177/0146167216666265 27677731

[B9] Czarna A. Z. Zajenkowski M. Dufner M. (2018). “How does it feel to be a narcissist? narcissism and emotions,” in *Handbook of Trait Narcissism. Key Advances, Research Methods, and Controversies*, eds Hermann A. Brunell A. B. Foster J. D. (Cham: Springer), 255–264.

[B10] Dufner M. Gebauer J. E. Sedikides C. Denissen J. J. (2019). Self-enhancement and psychological adjustment: a meta-analytic review. *Pers. Soc. Psychol. Rev.* 23 48–72. 10.1177/1088868318756467 29534642

[B11] Foster J. D. Brunell A. B. (2018). “Narcissism and romantic relationships,” in *Handbook of trait Narcissism. Key Advances, Research Methods, and Controversies*, eds Hermann A. Brunell A. B. Foster J. D. (Cham: Springer), 317–326. 10.1037/pspp0000113

[B12] Freud S. (1914). “On narcissism: an introduction,” in *The Standard Edition of the Complete Psychological Works of Sigmund Freud: On the History of the Psycho-analytic Movement, Papers on Metapsychology and Other Works*, ed. Strachey J. (London: The Hogarth Press and the Institute of Psycho-analysis).

[B13] Gignac G. E. Zajenkowski M. (2021). The frustrated narcissist: intelligence may reduce the chances of developing narcissistic rivalry. *Intelligence* 87:101556. 10.1016/j.intell.2021.101556

[B14] Grijalva E. Zhang L. (2015). Narcissism and self-insight: a review and meta-analysis of narcissists’ self-enhancement tendencies. *Pers. Soc. Psychol. B* 42 3–24. 10.1177/0146167215611636 26542339

[B15] Hayes A. F. (2015). An index and test of linear moderated mediation. *Multivar. Behav. Res.* 50 1–22. 10.1080/00273171.2014.962683 26609740

[B16] Howard M. C. Cogswell J. (2018). The “other” relationships of self-assessed intelligence: a meta-analysis. *J. Res. Pers.* 77 31–46. 10.1016/j.jrp.2018.09.006

[B17] Howes S. S. Kausel E. E. Jackson A. T. Reb J. (2020). When and why narcissists exhibit greater hindsight bias and less perceived learning. *J. Manage.* 46 1498152. 10.1177/0149206320929421

[B18] Kohut H. (1966). Forms and transformations of narcissism. *J. Am. Psychoanal. Ass.* 14 243–272. 10.1177/000306516601400201 5941052

[B19] Krizan Z. Herlache A. D. (2018). The narcissism spectrum model: a synthetic view of narcissistic personality. *Pers. Soc. Psychol. Rev.* 22 3–31. 10.1177/1088868316685018 28132598

[B20] Maltby J. Day L. Hall S. (2015). Refining trait resilience: identifying engineering, ecological, and adaptive facets from extant measures of resilience. *PLoS One* 10:e0131826. 10.1371/journal.pone.0131826 26132197 PMC4488934

[B21] Matthews G. Campbell S. E. Falconer S. Joyner L. Huggins J. Gilliland K. (2002). Fundamental dimensions of subjective state in performance settings: task engagement, distress and worry. *Emotion* 2 315–340. 10.1037/1528-3542.2.4.315 12899368

[B22] Meisel M. K. Ning H. Campbell W. K. Goodie A. S. (2016). Narcissism, overconfidence, and risk taking in U.S. and Chinese student samples. *J. Cross. Cult. Psychol.* 47 385–400. 10.1177/0022022115621968

[B23] Miller J. D. Hoffman B. J. Gaughan E. T. Gentile B. Maples J. Campbell W. K. (2011). Grandiose and vulnerable narcissism: a nomological network analysis. *J. Pers.* 79 1013–1042. 10.1111/j.1467-6494.2010.00711.x 21204843

[B24] Morf C. C. Rhodewalt F. (2001). Unraveling the paradoxes of narcissism: a dynamic self-regulatory processing model. *Psychol. Inq.* 12 177–196. 10.1207/S15327965PLI1204_1

[B25] Ng H. K. Cheung R. Y. H. Tam K. P. (2014). Unraveling the link between narcissism and psychological health: new evidence from coping flexibility. *Pers. Ind. Differ.* 70 7–10. 10.1016/j.paid.2014.06.006

[B26] Nunnally J. C. (1978). *Psychometric Theory*, 2nd Edn. New York, NY: McGraw Hill.

[B27] Papageorgiou K. A. Gianniou F. M. Wilson P. Moneta G. B. Bilello D. Clough P. J. (2019). The bright side of dark: exploring the positive effect of narcissism on perceived stress through mental toughness. *Pers. Ind. Differ.* 139 116–124. 10.1016/j.paid.2018.11.004

[B28] Paulhus D. L. Williams K. M. (2002). The dark triad of personality: narcissism, machiavellianism, and psychopathy. *J. Res. Pers.* 36 556–563. 10.3389/fpsyt.2019.00662 31607963 PMC6757332

[B29] Raven J. C. Court J. H. Raven J. (1983). *Manual for Raven’s Progressive Matrices and Vocabulary Scales (Section 4: Advanced Progressive Matrices).* London: H. K. Lewis.

[B30] Rhodewalt F. Morf C. C. (1998). On self-aggrandizement and anger: a temporal analysis of narcissism and affective reactions to success and failure. *J. Pers. Soc. Psychol.* 74:672. 10.1037//0022-3514.74.3.672 9523411

[B31] Rogoza R. Cieciuch J. Strus W. Baran T. (2019). Seeking a common framework for research on narcissism: an attempt to integrate the different faces of narcissism within the circumplex of personality metatraits. *Eur. J. Pers.* 33 437–455. 10.1002/per.2206

[B32] Rogoza R. Rogoza M. Wyszyńska P. (2016a). Polska adaptacja kwestionariusza NARQ [Polish adaptation of the NARQ]. *Polskie Forum Psychologiczne* 21 410–431. 10.14656/PFP20160306

[B33] Rogoza R. Wyszyńska P. Maćkiewicz M. Cieciuch J. (2016b). Differentiation of the two narcissistic faces in their relations to personality traits and basic values. *Pers. Ind. Differ.* 95 85–88. 10.1016/j.paid.2016.02.038

[B34] Rogoza R. Żemojtel-Piotrowska M. Kwiatkowska M. M. Kwiatkowska K. (2018). The bright, the dark and the blue face of narcissism: the spectrum of narcissism in its relations to the metatraits of personality, self-esteem, and nomological network of shyness, loneliness and empathy. *Front. Psychol.* 9:343. 10.3389/fpsyg.2018.00343 29593627 PMC5861199

[B35] Sabouri S. Gerber M. Sadeghi Bahmani D. Lemola S. Clough P. J. Kalak N. (2016). Examining dark triad traits in relation to mental toughness and physical activity in young adults. *Neuropsych. Dis. Treat.* 12 229–235. 10.2147/NDT.S97267 26869790 PMC4737324

[B36] Schmitt N. (1996). Uses and abuses of coefficient alphas. *Psychol. Assessment* 8 350–353. 10.1037/1040-3590.8.4.350

[B37] Sedikides C. Rudich E. A. Gregg A. P. Kumashiro M. Rusbult C. (2004). Are normal narcissists psychologically healthy: self-esteem matters. *J. Pers. Soc. Psychol.* 87 400–416. 10.1037/0022-3514.87.3.400 15382988

[B38] Sękowski M. Subramanian Ł Żemojtel-Piotrowska M. (2021). Are narcissists resilient? examining grandiose and vulnerable narcissism in the context of a three-dimensional model of resilience. *Curr. Psychol.* 1–9. 10.1007/s12144-021-01577-y

[B39] Wink P. (1991). Two faces of narcissism. *J. Pers. Soc. Psychol.* 61 590–597. 10.1037/0022-3514.61.4.590 1960651

[B40] Zajenkowski M. Dufner M. (2020). Why do narcissists care so much about intelligence? *Curr. Dir. Psychol. Sci.* 29 261–266. 10.1177/0963721420917152

[B41] Zajenkowski M. Gignac G. E. (2018). Why do angry people overestimate their intelligence? neuroticism as a suppressor of the association between trait-anger and subjectively assessed intelligence. *Intelligence* 70 12–21. 10.1016/j.intell.2018.07.003

[B42] Zajenkowski M. Matthews G. (2019). Intellect and openness differentially predict affect: perceived and objective cognitive ability contexts. *Pers. Ind. Differ.* 137 1–8. 10.1016/j.paid.2018.08.001

[B43] Zajenkowski M. Czarna A. Z. Szymaniak K. Dufner M. (2020a). What do highly narcissistic people think and feel about (their) intelligence? *J. Pers.* 88 703–718. 10.1111/jopy.12520 31654584

[B44] Zajenkowski M. Leniarska M. Jonason P. K. (2020c). Look how smart I am!: only narcissistic admiration is associated with inflated reports of intelligence. *Pers. Ind. Differ.* 165:100158. 10.1016/j.paid.2020.110158

[B45] Zajenkowski M. Jonason P. K. Leniarska M. Kozakiewicz Z. (2020b). Who complies with the restrictions to reduce the spread of COVID-19: personality and perceptions of the COVID-19 situation. *Pers. Ind. Differ.* 166:110199. 10.1016/j.paid.2020.110199 32565591 PMC7296320

